# Haptoglobin Preferentially Binds β but Not α Subunits Cross-Linked Hemoglobin Tetramers with Minimal Effects on Ligand and Redox Reactions

**DOI:** 10.1371/journal.pone.0059841

**Published:** 2013-03-29

**Authors:** Yiping Jia, Francine Wood, Paul W. Buehler, Abdu I. Alayash

**Affiliations:** Laboratory of Biochemistry and Vascular Biology, Center for Biologics Evaluation and Research, Food and Drug Administration, Bethesda, Maryland, United States of America; Universidad de Granada, Spain

## Abstract

Human hemoglobin (Hb) and haptoglobin (Hp) exhibit an extremely high affinity for each other, and the dissociation of Hb tetramers into dimers is generally believed to be a prerequisite for complex formation. We have investigated Hp interactions with native Hb, αα, and ββ cross-linked Hb (ααXLHb and ββXLHb, respectively), and rapid kinetics of Hb ligand binding as well as the redox reactivity in the presence of and absence of Hp. The quaternary conformation of ββ subunit cross-linking results in a higher binding affinity than that of αα subunit cross-linked Hb. However, ββ cross-linked Hb exhibits a four fold slower association rate constant than the reaction rate of unmodified Hb with Hp. The Hp contact regions in the Hb dimer interfaces appear to be more readily exposed in ββXLHb than ααXLHb. In addition, apart from the functional changes caused by chemical modifications, Hp binding does not induce appreciable effects on the ligand binding and redox reactions of ββXLHb. Our findings may therefore be relevant to the design of safer Hb-based oxygen therapeutics by utilizing this preferential binding of ββXLHb to Hp. This may ultimately provide a safe oxidative inactivation and clearance pathway for chemically modified Hbs in circulation.

## Introduction

Haptoglobin (Hp) has been extensively studied as an hemoglobin (Hb) scavenging protein due to its naturally high binding affinity towards extracellular Hb in plasma [1]. The Hb-Hp protein complex is removed from circulation through the CD163 scavenger receptor on the surface of peripheral blood and tissue monocytes and macrophages [2], [3], [4]. Free Hb is cleared to prevent cellular and tissue damage caused by oxidative reactions mediated by its heme iron. There has been renewed interest in Hp as a potential therapeutic for indications involving acute and chronic hemolysis, and as an adjuvant for acellular Hb-based oxygen therapeutics [5].

Hp is an acute phase plasma protein that exists in three primary phenotypes: Hp 1-1, Hp 2-1, and Hp 2-2 [6]. A Hp monomer consists of one α subunit and one β subunit linked via disulfide linkage, and one Hp monomer binds with one Hb αβ dimer. The Hp molecule consists of two types of α subunits, α1 (approximately 9 kDa) and α2 (approximately 16 kDa), and a single type of β subunit of Hp which weights approximately 45 kDa. The α1 subunit contains a single cysteine residue, while the α2 subunit contains an extra cysteine residue capable of forming multiple disulfide bonds. Therefore, Hp 1-1 is a dimer that consists of two α1 subunits and two β subunits. Hp 2-1 and Hp 2-2 contain α2 subunits to form polymers of different sizes and shapes. The Hb binding site on Hp has previously been mapped to the large β subunit [7].

It has been shown that all types of Hp bind oxygenated Hb almost irreversibly with the equilibrium dissociation constant (K_d_) reportedly as low as 10^−12^ M or 10^−15^ M [8], [9]. Purified Hb subunits can form much weaker complex with Hp, whereas the deoxyHb remains mostly as tetramers and appears to display no binding to Hp. The Hb dimer associates rapidly with Hp under oxygenated conditions, and Hb dimer formation appears to be essential to Hb-Hp binding [10].

In the absence of antioxidant enzymatic system within red blood cells, acellular Hb undergoes a series of oxidative reactions generating potentially toxic reactive oxygen species (ROS) such as superoxide (O_2_^•-^), hydrogen peroxide (H_2_O_2_). H_2_O_2_ is one of the well-examined Hb pro-oxidants that react with ferrous Hb (Fe^2+^) in a two-electron transfer process to produce the oxo-ferryl (Fe^4+^ = O^2−^) Hb species. When it reacts with met Hb (Fe^3+^), a protein radical (^•^HbFe^4+^ = O^2−^) is formed. Both of these higher oxidation states of Hb can cause redox side reactions, damaging Hb itself as well as other nearby proteins and lipids. These reactions have yet to be fully understood, but they are believed to originate from the highly reactive heme group. Radicals are thought to migrate via tyrosine residues to other amino acids [11]. As a consequence, irreversible oxidative modifications of specific amino acids, e.g., Trp15, Met55, Cys93, Cys112 and Tyr145 in the β subunits have been consistently observed in the presence of oxidants such as H_2_O_2_. Cross-linkages between Hb globin chains and heme which can trigger further oxidative reactivity have also been reported to occur [12].

Nitrite, another powerful oxidant, reacts with oxyHb in a complex process of multiple reaction steps influenced by Hb quaternary state intrinsic oxygen and ligand reactivity, and heme accessibility [13]. Nitrite also reacts with deoxyHb to generate metHb and NO. The latter enzyme-like function may have some physiological implications in the regulation of blood vessel dilation and blood pressure control. Hp has been demonstrated to enhance nitrite reductase activity of deoxyHb by approximately ten-fold, much like that of Hb dimers [14], [15]. Moreover recent studies have shown that Hp impedes radical formation, or stabilizes the damaging radicals formed in the presence of H_2_O_2_, which led us and others to explore the protective mechanisms of Hp more fully for possible therapeutic applications [5].

Hp interactions with several chemically modified Hbs have been previously evaluated using fluorometric methods and by surface plasmon resonance (SPR) analysis [16]. This analysis consistently revealed a strong affinity of Hp for Hbs, but a much lower affinity for internally cross-linked Hbs. Furthermore, these studies suggest that the αα subunits cross-linked Hbs may have more reduced affinity to Hp.

The Hp binding with modified and unmodified Hbs was examined closely in the present study using SEC-HPLC, native gel electrophoresis, and stopped-flow kinetic techniques. The functional effects of Hp binding on oxygen dissociation and ligand binding to internally cross-linked, non-dissociable tetrameric Hb were studied in comparison with that of unmodified Hb. The redox reactions of Hb with typical oxidants such as H_2_O_2_ and nitrite were assessed in order to determine the impact of specific chemical cross-linking and Hp binding of these reactions. Our results suggest that ββ subunit cross-linked tetrameric Hb retains substantial Hp binding capacity which does not alter ligand binding or redox reactions beyond that observed with chemical modification alone. This can have an important application in the design of safe cross-linked and/or polymerized hemoglobin-based blood substitutes. These results are also discussed in the light of a recently published crystal structure of the porcine Hp-Hb complex [17].

## Methods

### Materials

Human HbA was prepared as previously described via ammonium sulfate precipitation and anion-exchange FPLC, and stripped of endogenous organic phosphate cofactors [18]. Both αα subunits cross-linked Hb (ααXLHb) and ββ subunits cross-linked Hb (ββXLHb) by bis(3,5-dibromosalicyl)fumarate were prepared as previously described [19], and provided by Research Institutes of the United States Army (Washington, DC). The Hp sample containing primarily dimers (Hp 1-1) and to a lesser extent polymers (Hp 2-1, and Hp 2-2) was purified [20], and kindly provided by Bio Products Laboratory (BPL, Hertfordshire, UK). All chemicals and reagents were purchased from Sigma Aldrich (Saint Louis, Missouri) or Fisher Scientific (Pittsburgh, Pennsylvania) unless indicated otherwise, and gases were purchased from Roberts Oxygen Company, Inc. (Rockville, Maryland) or Matheson Tri Gas (Basking Ridge, New Jersey).

### HPLC and Gel Electrophoresis Analyses

Hp was mixed in excess with Hb (Hp:Hb, in a 2∶1 ratio) to allow for the complete binding of Hb to Hp. The extent of Hb binding to Hp was measured by size exclusion chromatography on a BioSep-SEC-S3000 column ((600 mm×7.5 mm), Phenomenex, Torrence CA) attached to a Waters 2535 quaternary gradient module and 2948 photodiode array detector (Waters Corporation, Milford, MA). Each run was normalized using a 50 µl injection loop. The mobile phase consisted of 50 mM potassium phosphate buffer, pH 7.4, at 22°C, pumped at a flow rate of 1.0 mL/minute over a 30 min run time. An injection loop flush step was performed prior to each run using 500 µL of mobile phase. The percentage of Hb bound to Hp was determined by dividing the area of Hb-Hp peaks (15–17 min elution time) at 405 nm by the total area of Hb containing peaks (15–21 min elution time) multiplied by 100.

The molecular weight compositions of the Hb, Hp, and Hb-Hp samples were assessed by native PAGE using an Invitrogen Novex® Minigel System and NativeMark™ Unstained Protein Standard (Carlsbad, CA). A 4–16% precast NativePAGE™ Novex Bis-Tris gel was employed, and each lane was loaded with 3–5 µL of sample solutions (Invitrogen NativePAGE™ sample prep kit) containing about 100 µM Hb and Hp in excess. The gel was run at 120 V for 90 minutes on a PowerEase 500 Power Supply. After electrophoresis, the gel was stained overnight with Coomassie blue G250 stain buffer, and then destained in water.

### Stopped-flow Fluorescence Measurement

The rapid reaction of Hb and Hp was monitored in an Applied Photophysics microvolume stopped-flow spectrophotometer (Leatherhead, UK) with a dead time of approximately 1.5 ms. Hp solutions (∼ 1 µM) were mixed in the stopped-flow with Hb solutions of various excess concentrations (up to 30 µM) in 50 mM sodium phosphate buffer, pH 7.4. The fluorescent change of the reaction was measured with an excitation wavelength at 285 nm, and a cutoff filter <360 nm for emission as a function of time. At least three time courses of Hp binding with Hbs were fitted to exponential equations to obtain the averaged pseudo-first-order rate constant for each reaction. Bimolecular rate constants were derived from the slope of the linear relationship of the apparent association rate constants as a function of Hb concentration.

### Rapid Kinetics of Ligand Reactions

The kinetics of oxygen dissociation from oxy Hb or the Hb-Hp complex, and binding of carbon monoxide (CO) to deoxy Hb or the Hb-Hp complex were measured in an Applied Photophysics microvolume stopped-flow instrument as previously described [21], [22]. Hb solutions (30 *µ*M in heme) were rapidly mixed with an equal volume of 1.5 mg/mL sodium dithionite, and the absorbance changes of the oxygen dissociation process were monitored at 437.5 nm in 50 mM Bis-Tris buffer at pH 7.4 at room temperature. The CO binding kinetics were measured at 437.5 nm in the same instrument and buffer containing freshly made 1.5 mg/mL sodium dithionite. The CO solution was prepared by saturating the degassed buffer with a flow of pre-washed CO gas. For each reaction, at least three kinetic traces were averaged and fit to exponential equations using the Marquardt−Levenberg fitting routines included in the Applied Photophysics software.

### Nitrite- and H_2_O_2_-induced Hb Oxidation

The spectral changes of the Hb reaction with nitrite were measured in an Agilent 8453 diode array spectrophotometer in the presence or absence of Hp. Rapid mixing methods using Applied Photophysics microvolume stopped-flow instrument were used to measure the kinetics of nitrite oxidation reaction of Hbs with and without Hp. Hb solutions (30 µM) were mixed with equal volumes of freshly prepared nitrite solution (6 mM) in potassium phosphate buffer, pH7.4, to initiate oxidation, and the absorbance changes were monitored as a function of time. The reaction time courses were recorded at a single wavelength, 577 nm, and repeated at least three times for each reaction condition.

The oxidation of met Hbs by excessive amount of H_2_O_2_ was performed under pseudo-first-order conditions using Applied Photophysics microvolume stopped-flow spectrophotometer with a diode array detector at 25°C, as described previously [23]. Ferric (met) Hb solutions (20 µM) with and without excess Hp in 0.5 mM Tris buffer, pH 7.4, were rapidly mixed with H_2_O_2_ solutions of increasing concentrations up to 100 mM. The absorbance spectral changes (at least 200 spectra) were recorded as a function of time. The whole set of spectral data were subjected to global curve fitting analysis (Applied Photophysics software) to derive the reaction rate constants of the oxidation reaction of met Hb to ferryl Hb. The second-order rate constants were obtained from the dependence of the apparent rate constants on H_2_O_2_ concentrations.

### The Measurement of Ferryl Hb Intermediate Formation and Decay

The intermediate formation of ferryl Hb in the reaction of Hb or Hb-Hp with H_2_O_2_ was detected by its reaction with sodium sulfide (Na_2_S), generating sulfonated Hb (sulfHb) that is spectrally detectable [24]. In a typical experiment, metHb (50 µM) was mixed with 1∶5 molar ratio of H_2_O_2_ in 50 mM potassium phosphate buffer, pH 7.4, to initiate the oxidative reaction in a cuvette monitored in an Agilent 8453 diode array spectrophotometer. After a 2 minute incubation time, Na_2_S (2 mM) was added to the reaction mixture and spectral changes between 450 nm and 700 nm were recorded for the conversion of ferryl Hb to sulfHb. The sulfHb concentrations were calculated using the extinction coefficient of 20.8 mM^−1^ cm^−1^ at 620 nm [24].

## Results

**Figure 1:**
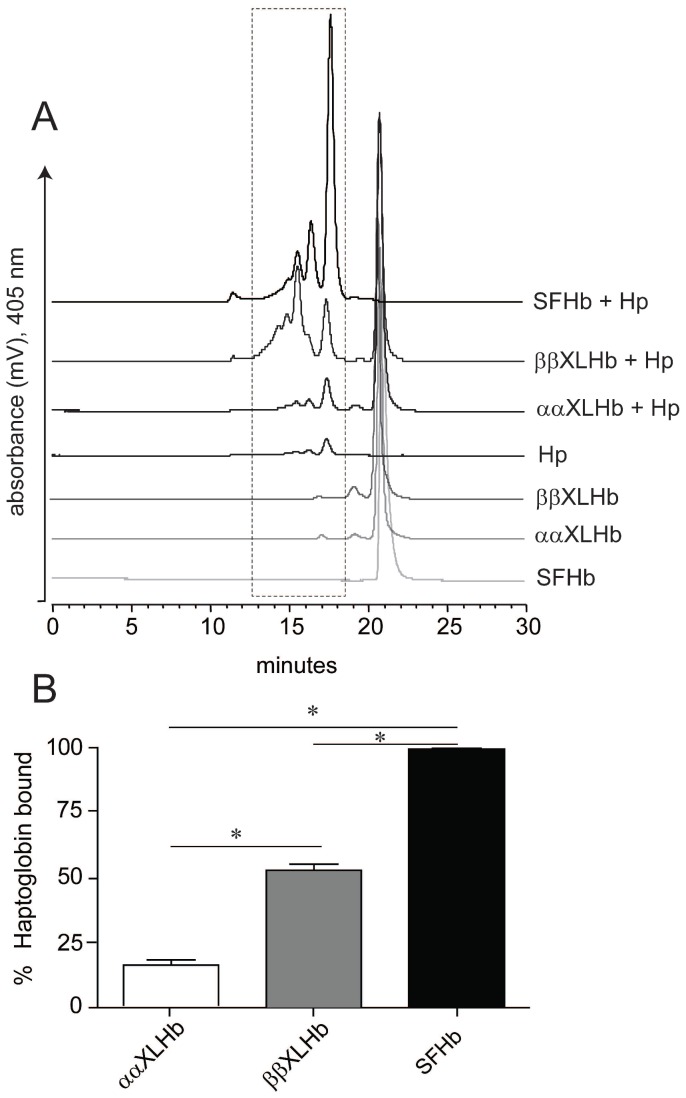
Analytical size exclusion chromatography of Hbs with or without Hp. A. Representative chromatograms of Hb, ααXLHb, and ββXLHb in the presence or absence of Hp. The absorbance was monitored at 405 nm for heme, and the peaks appearing between the elution time 13 and 19 minutes are Hb and Hp complexes. B. The percentage of Hp bound with Hb was determined and compared among ααXLHb, ββXLHb, and Hb based on baseline normalized area under curve for peaks within the boxed region of the eluting profiles (from Panel A). Statistical analysis was performed using a One-way analysis of variance with a Bonferroni’s Multiple comparison test to evaluate between group differences. Data are represented as mean areas +/− sem, significance was set at p<0.05. All analysis were performed using GraphPad Prism software.

Purified human Hb, ααXLHb and ββXLHb, and complexs of each protein with Hp were analyzed using both analytical size-exclusion chromatography (SEC) and native gel electrophoresis methods. SEC consistently revealed the major Hb peaks at the elution time of 20.9 min (monitored at 405 nm) in all Hb samples (Figure 1A). Three main peaks were observed between elution times 13 min and 18 min for the HbA sample with Hp, in agreement with the previously reported results [25]. The peak eluting at 17.3 minutes represents HbA bound to Hp 1-1, while Hb binding to polymeric species (Hp 2-2 or Hp 2-1) were observed with predominant peaks at 15.5 and 16.4 minutes, respectively. A slightly different elution profile with additional peaks eluting between 13 min and 16 min was observed for the mixture of ββXLHb and Hp, indicating further molecular association and the formation of larger molecular weight complexes. Only small peaks could be detected in the same elution time frame for the mixture of ααXLHb and Hp. Since Hp was added in excess, 99.6% ±0.4% purified human Hb sample was bound with Hp and no free Hb peak was observed. It was determined from peak % area calculations within the boxed region that ββXLHb preserved approximately 52.9% ±4.1% of the binding capacity with Hp relative to unmodified Hb, whereas ααXLHb and Hp showed minimal binding with less than 15.8% ±3.5% including non-specific bindings and low levels of contaminants (Figure 1B).

**Figure 2:**
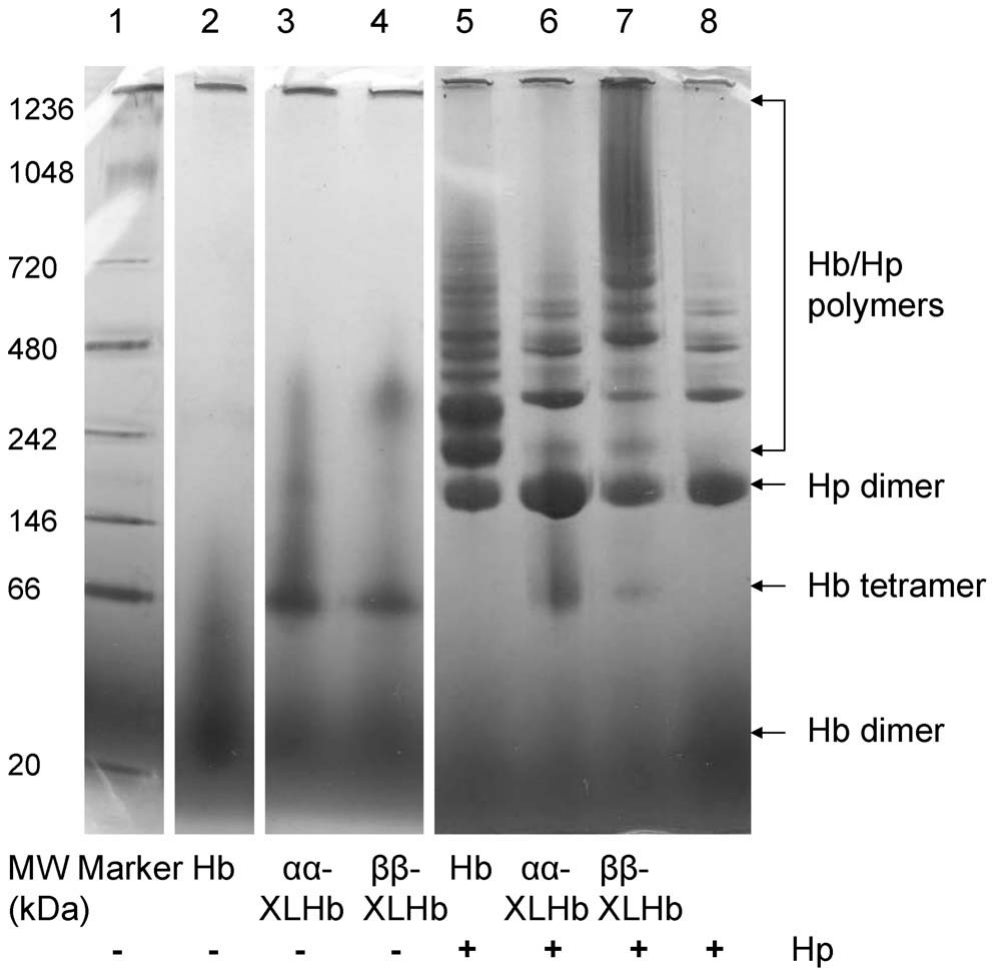
Native PAGE analysis of the Hb-Hp complexes. The samples of Hb, ααXLHb, and ββXLHb with and without Hp were analyzed in 4–16% Native PAGE under the same condition. The positions of Hb dimer and tetramer, Hp dimer and polymers, and the Hb and Hp complex formation on gels were indicated relative to that of molecular weight markers.

The SEC results were verified and confirmed on a native PAGE (Figure 2). The unmodified Hb was resolved largely into Hb dimers and some tetramers (Lane 2) as contrasted to the native gel molecular markers (Lane 1). The samples of ααXLHb (Lane 3) and ββXLHb (Lane 4) appeared primarily as tetramers, as a result of the chemical cross-linking and tetramer stabilization processes, with some minor protein impurities that may not be Hb species, and were not observed in the SEC-HPLC results. The Hp sample (Lane 8) was resolved primarily as Hp dimers (Hp 1-1), with some Hp polymer species, i.e., Hp 2-1 and Hp 2-2. In the presence of slight excess Hp (Lane 5), the formation of additional protein bands, and the disappearance of free Hb indicated the binding between Hp and unmodified Hb. The Hp dimers, and Hp polymers composed of Hp 2-1 and Hp 2-2 can form multiple protein complexes with native Hb to give extra bands. Conversely, relatively few new protein bands were observed in the case of the ααXLHb and Hp sample (Lane 6), which could represent a merge of the two samples run separately in Lanes 3 and 8. However, protein complexes of high molecular weight polymers formed between Hp and ββXLHb can be seen (Lane 7) with some ββXLHb tetramer also visible. This observation suggests a moderate binding capability, consistent with the SEC data. In addition, Hb dimers bind to Hp with strong monovalent binding characteristics, and ββXLHb binds to Hp with polyvalent binding characteristics. Our previous data suggest that ββXLHb polyvalent binding with Hp leads to a time dependent gelation phenomenon [16]. These binding characteristics also contribute to the observation of multiple band patterns observed in lanes 5 and 7.

**Figure 3:**
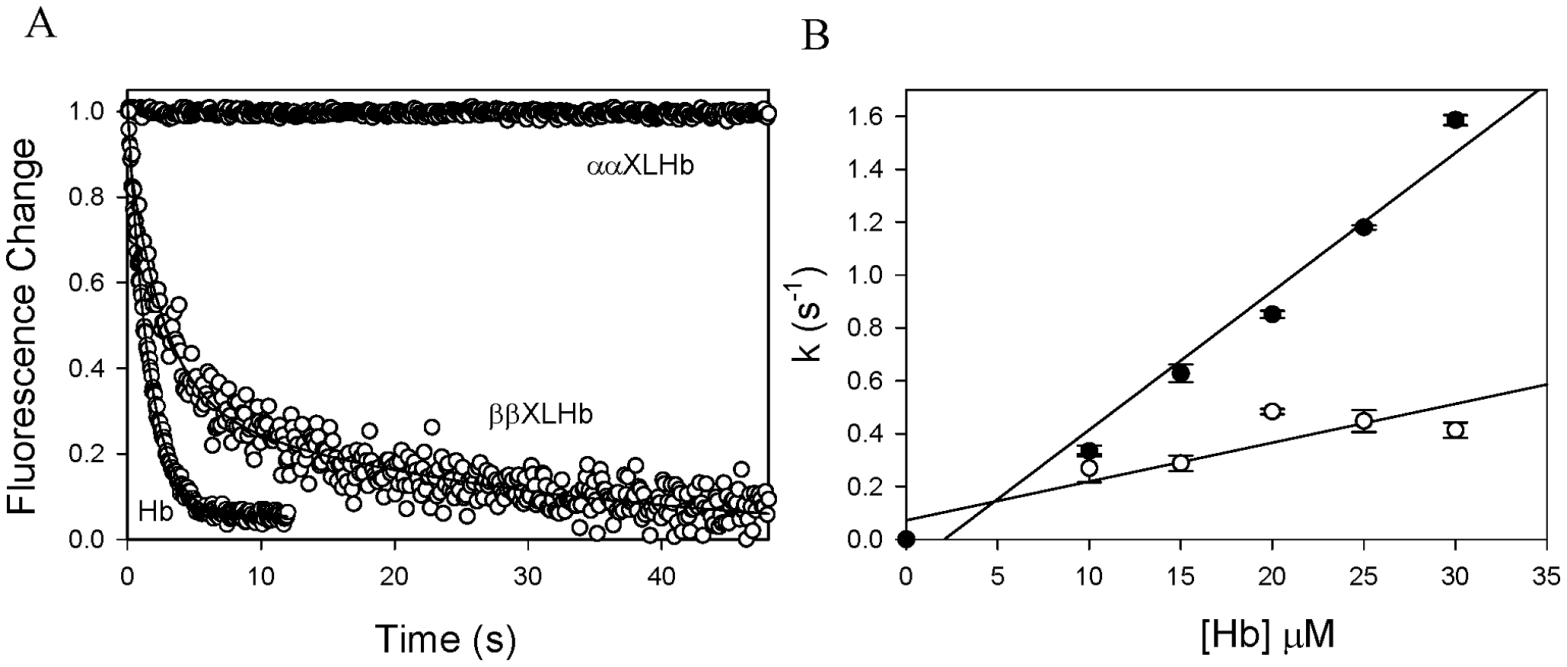
Rapid kinetics of Hp reaction with Hb measured by fluorescence emission change. A. The time courses of Hp binding with Hb (15 µM) were fitted to exponential equations to obtain the pseudo-first-order rate constants for Hb, ααXLHb, and ββXLHb. B. The second order rate constants of Hp binding with native Hb (close circle) and ββXLHb (open circle) were derived from the Hb concentration dependence of the obtained rate constants.

Hb binding to Hp is accompanied by the quenching of Hp intrinsic fluorescence as previously reported [10], [26]. We measured the kinetics of Hb and Hp binding by monitoring fluorescence changes in a rapid mixing stopped-flow spectrophotometer equipped with a fluorescence detector. Representative time courses of Hp (1 µM) association with 15 µM Hb, ααXLHb, or ββXLHb under pseudo first order conditions are shown in Figure 3A. The interaction of Hb and Hp was fitted to a single exponential equation. No reaction was observed between ααXLHb and Hp under the same experimental conditions. In contrast, ββXLHb and Hp displayed a relatively slower reaction followed by a much slower second kinetic phase, possibly due to polymer formation. These results are consistent with the observations using SEC and native PAGE studies of unmodified and modified Hbs binding with Hp. The Hb concentration dependence of the apparent association rate constants as shown in Figure 3B revealed that ββXLHb and Hb bind to Hp under our experimental conditions with bimolecular rate constants at about 0.015±0.004 µM^−1^s^−1^ and 0.052±0.004 µM^−1^s^−1^, respectively. The latter value is close to that reported previously for human Hb binding to Hp type 1–1 [27].

**Figure 4:**
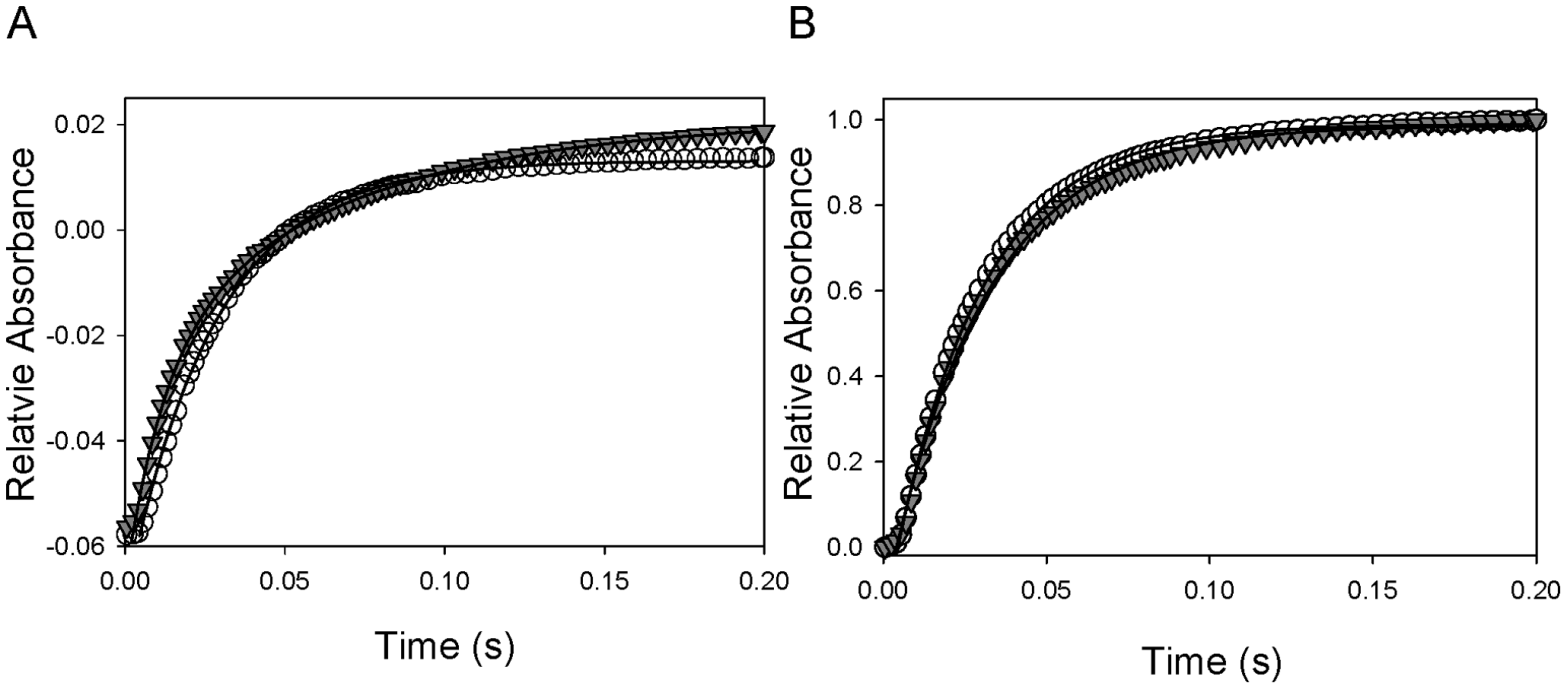
Stopped-flow kinetics of oxygen dissociation from Hbs in the presence and absence of Hp. A. The time courses of oxygen dissociation from HbA (open cirlce), and the Hb and Hp complex (gray triangle) mixed with 1.5 mg/mL sodium dithionite (Na_2_S_2_O_4_) were plotted for comparison. B. The time courses of oxygen dissociation from ββXLHb (open circle), and the ββXLHb complex with Hp (gray triangle) were illustrated.

The effects of Hp binding with unmodified Hb and cross-linked Hb tetramers on the rapid kinetics of ligand association and dissociation were evaluated. Figure 4A shows the time courses of O_2_ dissociation from Hb and the Hp-Hb complex. The binding of Hp clearly altered the overall time course from a single exponential process (34 s^−1^) to a biphasic reaction with apparent rate constants of 69 s^−1^ and 14 s^−1^ based on the nonlinear least squares curve fitting. In contrast, identical O_2_ dissociation time courses were recorded for ββXLHb bound or not bound to Hp (Figure 4B), with no appreciable difference in derived rate constants (Table 1).

**Table 1:** Ligand binding kinetic parameters of HbA and ββXLHb in the presence and absence of Hp.

	k_off_ (s^−1^)	k_on,_ _co_ (µM^−1^s^−1^)
HbA	34	0.22
HbA+Hp	69 (k_fast_)	∼ 2 (k_fast_)
	14 (k_slow_)	0.8 (k_slow_)
ββXLHb	36.7	0.33
ββXLHb+Hp	33.2	0.36
ααXLHb	45.6	0.21

**Figure 5:**
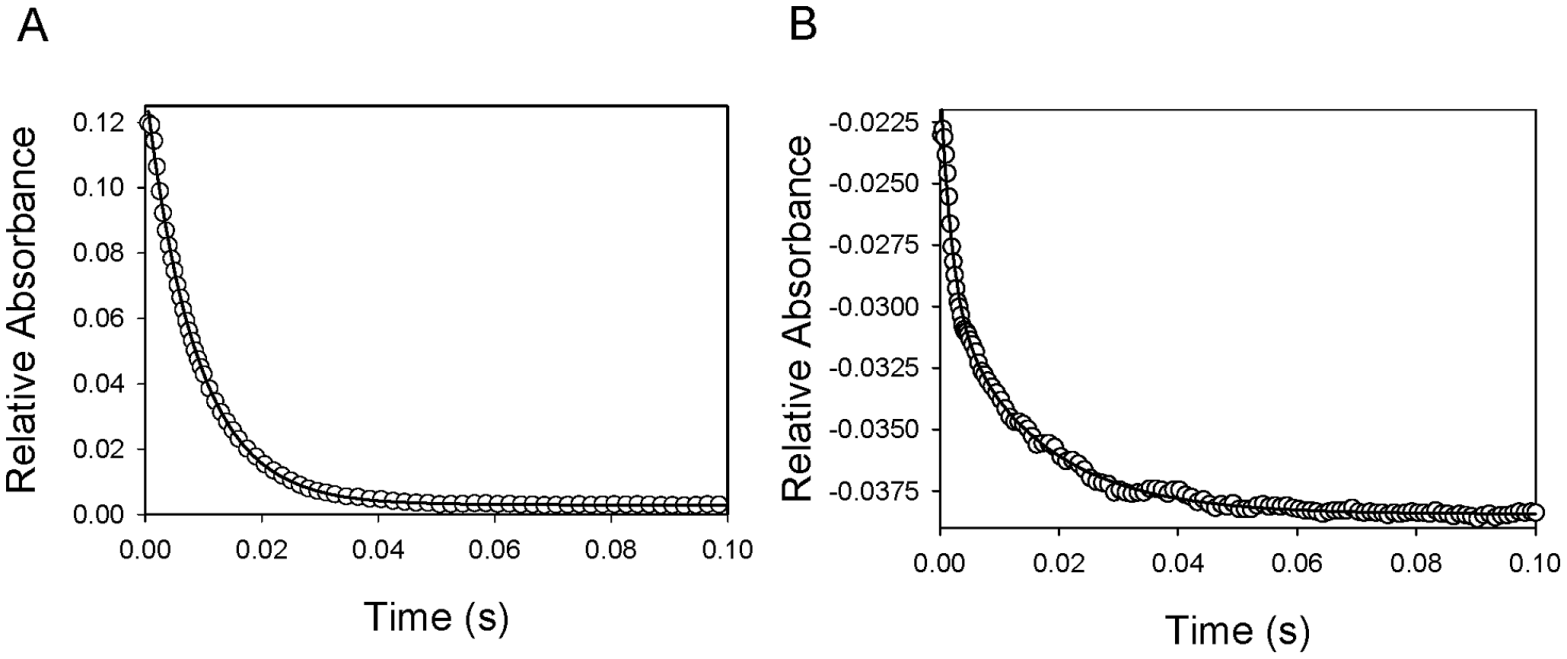
Stopped-flow kinetics of CO ligand association with HbA in the presence and absence of Hp. A. Representative time course of CO (500 µM after mixing) binding with HbA was fitted to single exponential equation by non-linear least squares regression analysis. B. Representative time course of CO (250 µM after mixing) binding with the HbA and Hp complex was biphasic, and fitted to double exponentials to derive apparent association rate constants.

**Figure 6:**
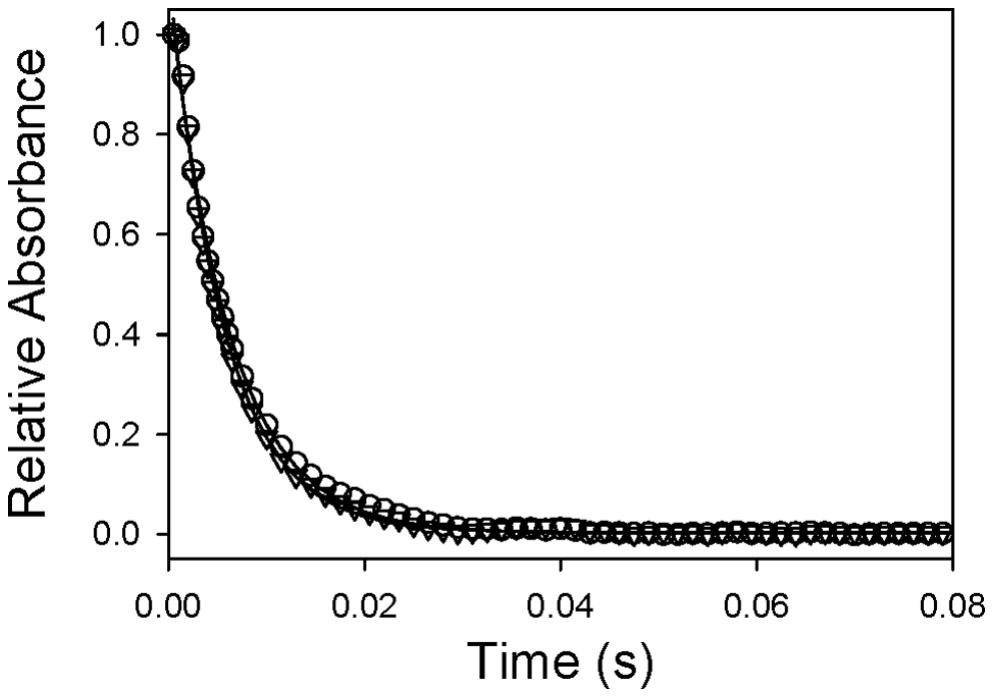
Stopped-flow kinetics of CO association with ββXLHb in the presence and absence of Hp. Representative time courses of CO (500 µM after mixing) binding with ββXLHb without (open circle) or with (open triangle) Hp were plotted for comparison. Both traces were fitted to single exponential equation by non-linear least squares regression analysis.

Figure 5A and 5B illustrate representative time courses of CO association with Hb in the absence and presence of Hp, respectively. The Hb reaction with CO is typically a single exponential process, whereas Hb-Hp complexes exhibit kinetic heterogeneity, including an additional fast phase with approximately 10 times larger second-order rate constant under the same experimental conditions (Table 1). In contrast, the recorded time courses of CO reaction with ββXLHb and the complex of ββXLHb and Hp were identical as depicted in Figure 6, and no significant differences in the derived second-order rate constants were observed (Table 1).

**Figure 7:**
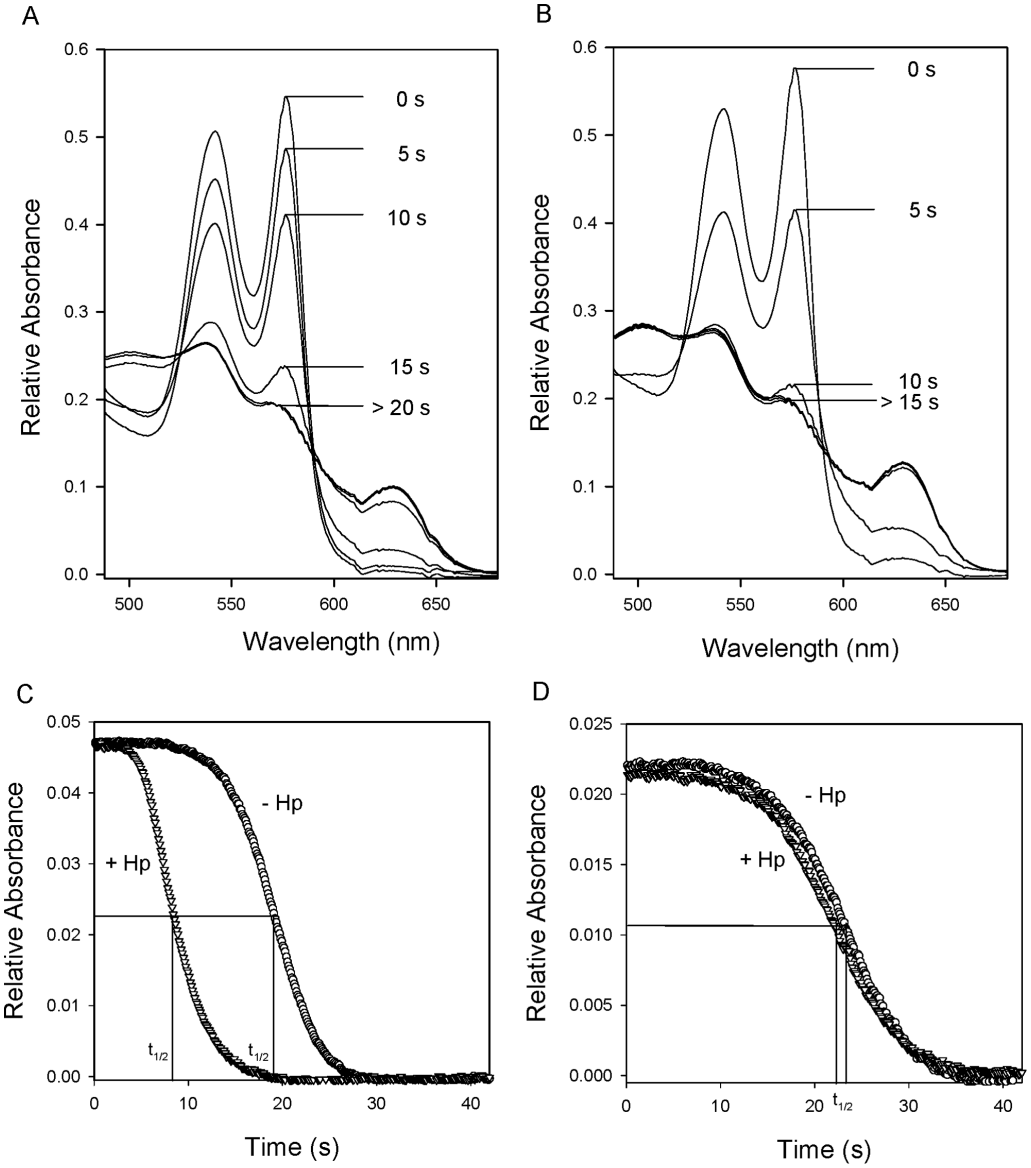
Nitrite reaction with oxy HbA and ββXLHb in the presence and absence of Hp. The spectral changes over time were measured in a spectrophotometer for the reaction of oxy HbA (30 µM) and nitrite (6 mM) in the absence (A) or the presence (B) of excess Hp. The kinetics of oxy HbA (C) and ββXLHb (D) reacting with freshly prepared nitrite as measured by rapid mixing, and the absorbance change was monitored at 577 nm. The time courses of the complex nitrite-induced Hb oxidation processes in the absence (open circle) and presence (open triangle) of Hp were illustrated, and the half times of the reaction under the same conditions were derived and listed in Table 2.

The effects of Hp binding on Hb oxidative reactions were examined using nitrite and H_2_O_2_ as oxidants. It is known that oxygenated Hb reacts with nitrite following a complex process to form metHb and nitrate as end products [28], [29], [30]. Figure 7A & 7B present the progressive spectral changes from oxy to met Hb over the reaction time of nitrite and native Hb with and without Hp. Fewer intermediate spectra and faster reaction were observed in the presence of Hp (Figure 7B). Figure 7C shows the typical auto-accelerating time course by monitoring the absorbance change of the oxy Hb and nitrite reaction to completion. Hp binding shifted the curve to the left, enhancing the auto-accelerating reaction of nitrite with the Hb and the Hb-Hp complex and changing the reaction half-time from 19.0 s to 8.4 s under our experimental conditions. Conversely, nitrite-induced oxidation of ββXLHb was not altered by Hp (Figure 7D), and both reactions exhibited similar auto-accelerating process with a half-time of 22–23 s^−1^ under the same conditions (Table 2).

**Table 2:** Hb redox reaction parameters in the presence and absence of Hp.

	Nitrite oxidation (t_1/2_) (s)	H_2_O_2_ oxidation (M^−1^s^−1^)	SulfHb initial rate (µM/min)
HbA	19.0	43.1	1.01
HbA+Hp	8.4	41.5	0.26
ββXLHb	22.9	44.4	0.32
ββXLHb+Hp	22.1	45.2	0.36

Hp binding was previously shown to have no effect on Hb pseudoperoxidase activity, the conversion of met to ferryl Hb with H_2_O_2_ consumption [2]. The absorbance changes of the formation of ferryl Hb were measured under pseudo first-order conditions in a rapid mixing stopped-flow instrument using a photodiode array detector. The progressive spectral changes in the Soret and visible regions were subjected to global kinetic analysis to de-convolute and reconstruct the spectra of met and ferryl Hbs and to derive the reaction rate constants. The observed rates were a linear function of the H_2_O_2_ concentrations, resulting in the second-order rate constants for this oxidation reaction from met to ferryl Hb (Table 2). No appreciable differences of the H_2_O_2_ oxidation rates were obtained for either Hb or ββXLHb in the absence or presence of Hp.

**Figure 8:**
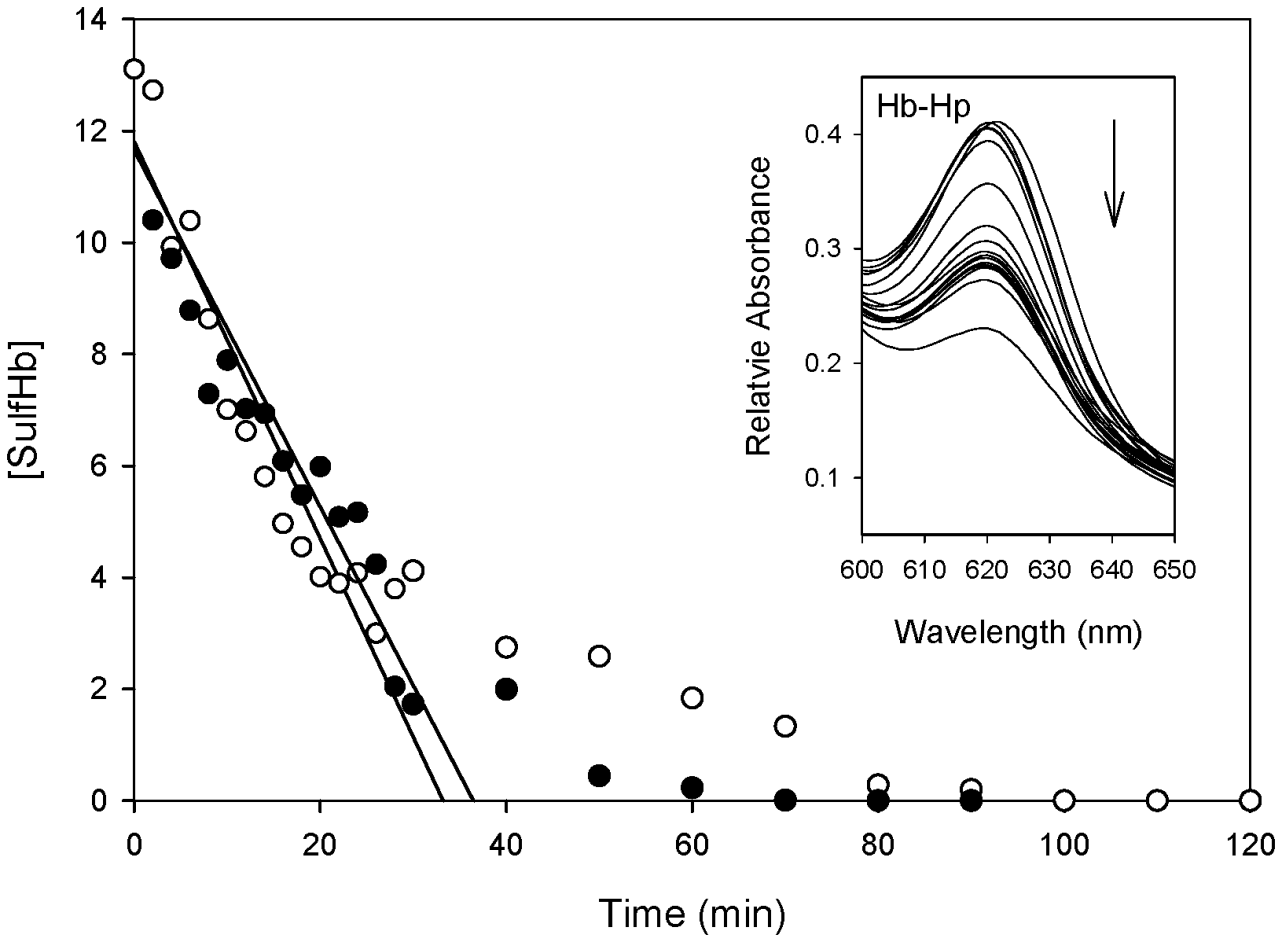
Hydrogen peroxide-induced Ferryl/sulf ββXLHb formation in the presence and absence of Hp. Ferryl Hb formation and stabilization measured as sulfHb concentrations in the reaction of metHb and H_2_O_2_. The close (•) and open (○) circles in the graph represent the sulfHb concentrations at reaction times of H_2_O_2_-induced metHb oxidation in the absence and presence of excess Hp, respectively. The inset shows typical spectral changes of resultant sulfHb at approximate 620 nm as a function of time. The approximate initial reaction rates were obtained and listed in Table 2.

The formation of ferryl Hb is followed by a process of auto-reduction to generate metHb which completes a pseudoperoxidase cycle [31]. The transient formation of ferryl Hb can be captured prior to its decay to metHb by adding sodium sulfide to convert the ferryl species to a spectrally more distinct and stable sulfHb species. It was shown in our previous report [15] that although the ferryl Hb concentration reached similar initial levels, the decay of ferryl Hb was attenuated by Hp binding. Figure 8 depicts the kinetic stabilization of ferryl ββXLHb as result of Hp binding in comparison with that of unmodified Hb. The levels of the initial ferryl ββXLHb formation were comparable to that of Hb, and progressive spectral changes focusing on the sulfHb peak at 620 nm is shown as a function of time in the inset. These Data demonstrate that the ferryl form of ββXLHb decayed similarly with initial rates of 0.32 µM/min and 0.36 µM/min in the absence and presence of Hp (Table 2). Although Hp reduced the initial rates from 1.01 µM/min to 0.26 µM/min for wild type Hb, no kinetic effects on ferryl Hb decay were detected in Hp complexed with ββXLHb.

## Discussion

The interaction between Hb and Hp is extremely strong and almost irreversible, equivalent to that of antigen-antibody interactions. Hb dimers have long been regarded as the only Hb molecular form that binds with Hp. However, non-dissociable chemically cross-linked Hbs have been observed to bind with Hp [16], [32]. The nature of chemically modified Hb and Hp protein complex formation may provide valuable insights into the molecular pathway of Hb clearance under physiological conditions, and may provide a better understanding of Hp mediated attenuation of Hb oxidative reactions [25].

The chemical modifications of Hb used in this study are site-specific in which the intramolecular reagent, bis(3,5-dibromosalicyl)fumarate was used to either cross-link the two Lys99 residues in α subunits of the deoxy or the two Lys82 residues in β subunits of the oxy forms of human Hb, resulting in ααXLHb and ββXLHb respectively [33], [34], [35], [36]. These structural changes produce stable, low oxygen (T-state) and high oxygen (R-state) affinity Hbs for transfusion purposes in animals and in humans [37], [38]. We reasoned that Hb quaternary conformations of ααXLHb and ββXLHb differ sufficiently to restrain ααXLHb and Hp interaction, but allow ββXLHb enough flexibility in its R conformation to serve as points of contact with Hp.

Our protein binding analyses clearly showed that the β subunit cross-linked Hb tetramers bind Hp, whereas tetrameric Hb cross-linked between α subunits has minimal interaction with Hp. SEC-HPLC revealed that approximately 50% of ββXLHb formed complex with Hp compared to 100% complex formation with unmodified Hb and Hp and that ββXLHb appeared to form larger sized complexes. Polymer formation is likely a result of ββXLHb divalent interaction with Hp, in which binding can occur at dimeric sites on the stabilized Hb tetramer [32]. The binding patterns of native Hb and ββXLHb with Hp were resolved by non-denaturing electrophoresis gel analysis which confirmed the SEC-HPLC results. Our rapid mixing analyses indicated that ββXLHb reacts with Hp at a rate that is about 4 fold slower than that of the native Hb. This was followed in the case of ββXLHb by a much slower second kinetic phase, which can be attributed to polymer formation.

We have characterized the impact of Hp interactions on the ligand binding reactions of chemically modified Hbs in order to understand the extent of the structural perturbation introduced by Hp. We also reported that both oxygen equilibrium binding and the kinetics of CO association and oxygen dissociation were altered after complex formation. In addition, we find that chemical modification of ααXLHb significantly decreases oxygen equilibrium binding affinity while increasing the oxygen off rate, whereas ββXLHb exhibits a higher oxygen affinity and CO on rate. Our equilibrium (data not shown) and kinetic parameters determined for the ββXLHb and Hp complex are similar to that of the uncomplexed ββXLHb. Interestingly, it appears that the complex formation between the R-state ββXLHb and Hp did not perturb its heme group reaction environment. Therefore, it is possible to alter Hb structure, promote Hp binding and retain desired ligand reaction properties.

In agreement with previous reports, our results showed that Hp does not change the intrinsic reactivity of Hb with H_2_O_2_, but largely increased ferryl Hb stability. It was previously shown that heme accessibility plays a major role in determining the reaction rate with nitrite [13]. We observed enhanced nitrite oxidation of native Hb in the presence of Hp due to Hb dimerization and potentially heme accessibility. Although chemical modification typically enhances redox reactivity of modified Hb deviations, Hp association with ββXLHb did not cause any additional H_2_O_2_ and nitrite reactivity changes. This suggests that modified Hb can bind Hp and retain heme reactivities.

Our results are in agreement with a recent crystal structure analysis of the porcine Hb and Hp complex [17]. Although porcine blood was used as source, porcine and human Hb and Hp exhibit 82% homology by sequence alignment, and the same dumbbell shape with two serine protease domains connected by two complement control protein (CCP) domains. The serine protease domain contains several loops exposed on the surface and the amino-terminal region that were determined to constitute the Hb-binding site. Hp interacts with both Hb α and β subunits, and amino acid residues in helix C, G and FG were determined as the primary sites, but also as Hb dimer interface in the formation of tetramer. These structural data also reveal that the conformation of deoxygenated Hb dimers does not promote Hp binding. Remarkably, these data also show that Hb-Hp interaction originates from an initial complex between C terminus of Hb α subunit and the active serine protease of Hp. This may explain the preferred binding to Hp we observed, as α subunits are more surface exposed due to the cross linkage of the β subunits within ββXLHb.

In summary, although Hb binds to Hp with a high affinity via Hb αβ dimerization, non-dissociable Hb tetramers may also form protein complex with Hp. Hb tetramers cross-linked between two β subunits retain an R-state like conformation and display much higher Hp affinity than that of α subunit cross-linked Hb tetramers. Because the binding of ββXLHb to Hp like unmodified Hb retains its ability to decrease the propagation of damaging ferryl radicals, site specific cross-linking of the β subunits may provide a basis for an improved design of Hb-based oxygen therapeutics.

## References

[1] Nielsen MJ, Petersen SV, Jacobsen C, Thirup S, Enghild JJ, et al (2007) A unique loop extension in the serine protease domain of haptoglobin is essential for CD163 recognition of the haptoglobin-hemoglobin complex. J Biol Chem 282: 1072–1079. 17102136 10.1074/jbc.M605684200

[2] Buehler PW, Abraham B, Vallelian F, Linnemayr C, Pereira CP, et al (2009) Haptoglobin preserves the CD163 hemoglobin scavenger pathway by shielding hemoglobin from peroxidative modification. Blood 113: 2578–2586. 19131549 10.1182/blood-2008-08-174466

[3] Schaer CA, Vallelian F, Imhof A, Schoedon G, Schaer DJ (2007) CD163-expressing monocytes constitute an endotoxin-sensitive Hb clearance compartment within the vascular system. J Leukoc Biol 82: 106–110. 17460152 10.1189/jlb.0706453

[4] Kristiansen M, Graversen JH, Jacobsen C, Sonne O, Hoffman HJ, et al (2001) Identification of the haemoglobin scavenger receptor. Nature 409: 198–201. 11196644 10.1038/35051594

[5] Alayash AI (2011) Haptoglobin: Old protein with new functions. Clin Chim Acta 412: 493–498. 21159311 10.1016/j.cca.2010.12.011

[6] Levy AP, Asleh R, Blum S, Levy NS, Miller-Lotan R, et al (2010) Haptoglobin: basic and clinical aspects. Antioxid Redox Signal 12: 293–304. 19659435 10.1089/ars.2009.2793

[7] Valette I, Waks M, Wejman JC, Arcoleo JP, Greer J (1981) Haptoglobin heavy and light chains. Structural properties, reassembly, and formation of minicomplex with hemoglobin. J Biol Chem 256: 672–679. 7451467

[8] Polticelli F, Bocedi A, Minervini G, Ascenzi P (2008) Human haptoglobin structure and function–a molecular modelling study. FEBS J 275: 5648–5656. 18959750 10.1111/j.1742-4658.2008.06690.x

[9] Hwang PK, Greer J (1980) Interaction between hemoglobin subunits in the hemoglobin. haptoglobin complex. J Biol Chem 255: 3038–3041. 7358726

[10] Nagel RL, Gibson QH (1971) The binding of hemoglobin to haptoglobin and its relation to subunit dissociation of hemoglobin. J Biol Chem 246: 69–73. 5541775

[11] Cooper CE, Schaer DJ, Buehler PW, Wilson MT, Reeder BJ, et al.. (2012) Haptoglobin Binding Stabilizes Hemoglobin Ferryl Iron and the Globin Radical on Tyrosine beta145. Antioxid Redox Signal [Epub ahead of print]. 10.1089/ars.2012.4547 PMC3638561 22702311

[12] Jia Y, Buehler PW, Boykins RA, Venable RM, Alayash AI (2007) Structural basis of peroxide-mediated changes in human hemoglobin: a novel oxidative pathway. J Biol Chem 282: 4894–4907. 17178725 10.1074/jbc.M609955200

[13] Bonaventura C, Henkens R, De Jesus-Bonilla W, Lopez-Garriga J, Jia Y, et al (2010) Extreme differences between hemoglobins I and II of the clam Lucina pectinalis in their reactions with nitrite. Biochim Biophys Acta 1804: 1988–1995. 20601225 10.1016/j.bbapap.2010.06.016 PMC2931271

[14] Roche CJ, Dantsker D, Alayash AI, Friedman JM (2012) Enhanced nitrite reductase activity associated with the haptoglobin complexed hemoglobin dimer: functional and antioxidative implications. Nitric Oxide 27: 32–39. 22521791 10.1016/j.niox.2012.04.002 PMC3580216

[15] Banerjee S, Jia Y, Parker Siburt CJ, Abraham B, Wood F, et al (2012) Haptoglobin alters oxygenation and oxidation of hemoglobin and decreases propagation of peroxide-induced oxidative reactions. Free Radic Biol Med 53: 1317–1326. 22841869 10.1016/j.freeradbiomed.2012.07.023

[16] Buehler PW, Vallelian F, Mikolajczyk MG, Schoedon G, Schweizer T, et al (2008) Structural stabilization in tetrameric or polymeric hemoglobin determines its interaction with endogenous antioxidant scavenger pathways. Antioxid Redox Signal 10: 1449–1462. 18522492 10.1089/ars.2008.2028

[17] Andersen CB, Torvund-Jensen M, Nielsen MJ, de Oliveira CL, Hersleth HP, et al (2012) Structure of the haptoglobin-haemoglobin complex. Nature 489: 456–459. 22922649 10.1038/nature11369

[18] Bonaventura C, Cashon R, Bonaventura J, Perutz M, Fermi G, et al (1991) Involvement of the distal histidine in the low affinity exhibited by Hb Chico (Lys beta 66–-Thr) and its isolated beta chains. J Biol Chem 266: 23033–23040. 1744099

[19] Highsmith FA, Driscoll CM, Chung BC, Chavez MD, Macdonald VW, et al (1997) An improved process for the production of sterile modified haemoglobin solutions. Biologicals 25: 257–268. 9324994 10.1006/biol.1997.0096

[20] Dalton J, Podmore A, Kumpalume P (2009) Method for the isolation of haptoglobin. Patent US2009/0281282.

[21] Antonini E, Brunori M (1971) Hemoglobin and myoglobin in their reactions with ligands. Amsterdam: North-Holland Publishing Company.

[22] Jia Y, Wood F, Menu P, Faivre B, Caron A, et al (2004) Oxygen binding and oxidation reactions of human hemoglobin conjugated to carboxylate dextran. Biochim Biophys Acta 1672: 164–173. 15182936 10.1016/j.bbagen.2004.03.009

[23] Nagababu E, Ramasamy S, Rifkind JM, Jia Y, Alayash AI (2002) Site-specific cross-linking of human and bovine hemoglobins differentially alters oxygen binding and redox side reactions producing rhombic heme and heme degradation. Biochemistry 41: 7407–7415. 12044174 10.1021/bi0121048

[24] Carrico RJ, Peisach J, Alben JO (1978) The preparation and some physical properties of sulfhemoglobin. J Biol Chem 253: 2386–2391. 204647

[25] Boretti FS, Buehler PW, D’Agnillo F, Kluge K, Glaus T, et al (2009) Sequestration of extracellular hemoglobin within a haptoglobin complex decreases its hypertensive and oxidative effects in dogs and guinea pigs. J Clin Invest 119: 2271–2280. 19620788 10.1172/JCI39115 PMC2719941

[26] Chiancone E, Alfsen A, Ioppolo C, Vecchini P, Agro AF, et al (1968) Studies on the reaction of haptoglobin with haemoglobin and haemoglobin chains. I. Stoichiometry and affinity. J Mol Biol 34: 347–356. 5760461 10.1016/0022-2836(68)90258-1

[27] Cohen-Dix P, Noble RW, Reichlin M (1973) Comparative binding studies of the hemoglobin-haptoglobin and the hemoglobin-antihemoglobin reactions. Biochemistry 12: 3744–3751. 4788311 10.1021/bi00743a025

[28] Kosaka H, Imaizumi K, Tyuma I (1982) Mechanism of autocatalytic oxidation of oxyhemoglobin by nitrite. An intermediate detected by electron spin resonance. Biochim Biophys Acta 702: 237–241. 6282334 10.1016/0167-4838(82)90508-8

[29] Kosaka H, Tyuma I (1982) Production of superoxide anion by N,N-bis(2-hydroxyethyl)-iminotris(hydroxymethyl)methane buffer during oxidation of oxyhemoglobin by nitrite and effect of inositol hexaphosphate on the oxidation. Biochim Biophys Acta 709: 187–193. 6295490 10.1016/0167-4838(82)90460-5

[30] Lissi E (1998) Autocatalytic oxidation of hemoglobin by nitrite: a possible mechanism. Free Radic Biol Med 24: 1535–1536. 9641272 10.1016/s0891-5849(98)00004-5

[31] Alayash AI, Ryan BA, Eich RF, Olson JS, Cashon RE (1999) Reactions of sperm whale myoglobin with hydrogen peroxide. Effects of distal pocket mutations on the formation and stability of the ferryl intermediate. J Biol Chem 274: 2029–2037. 9890961 10.1074/jbc.274.4.2029

[32] Benesch RE, Ikeda S, Benesch R (1976) Reaction of haptoglobin with hemoglobin covalently cross-linked between the alpha beta dimers. J Biol Chem 251: 465–470. 1245483

[33] Chatterjee R, Walder RY, Arnone A, Walder JA (1982) Mechanism for the increase in solubility of deoxyhemoglobin S due to cross-linking the beta chains between lysine-82 beta 1 and lysine-82 beta 2. Biochemistry 21: 5901–5909. 6817783 10.1021/bi00266a027

[34] Vandegriff KD, Le Tellier YC, Winslow RM, Rohlfs RJ, Olson JS (1991) Determination of the rate and equilibrium constants for oxygen and carbon monoxide binding to R-state human hemoglobin cross-linked between the alpha subunits at lysine 99. J Biol Chem 266: 17049–17059. 1910038

[35] Vandegriff KD, Medina F, Marini MA, Winslow RM (1989) Equilibrium oxygen binding to human hemoglobin cross-linked between the alpha chains by bis(3,5-dibromosalicyl) fumarate. J Biol Chem 264: 17824–17833. 2808353

[36] Walder JA, Zaugg RH, Walder RY, Steele JM, Klotz IM (1979) Diaspirins that cross-link beta chains of hemoglobin: bis(3,5-dibromosalicyl) succinate and bis(3,5-dibromosalicyl) fumarate. Biochemistry 18: 4265–4270. 486423 10.1021/bi00587a001

[37] Bucci E, Razynska A, Urbaitis B, Fronticelli C (1989) Pseudo cross-link of human hemoglobin with mono-(3,5-dibromosalicyl)fumarate. J Biol Chem 264: 6191–6195. 2495279

[38] Snyder SR, Welty EV, Walder RY, Williams LA, Walder JA (1987) HbXL99 alpha: a hemoglobin derivative that is cross-linked between the alpha subunits is useful as a blood substitute. Proc Natl Acad Sci U S A 84: 7280–7284. 3478694 10.1073/pnas.84.20.7280 PMC299276

